# Widespread Selection Across Coding and Noncoding DNA in the Pea Aphid Genome

**DOI:** 10.1534/g3.113.005793

**Published:** 2013-06-01

**Authors:** Ryan D. Bickel, Joseph P. Dunham, Jennifer A. Brisson

**Affiliations:** * University of Nebraska, School of Biological Sciences, Lincoln, Nebraska 68588; † University of Southern California, Molecular & Computational Biology, Los Angeles, California 90089

**Keywords:** pea aphid, Acyrthosiphon, population genomics, sex chromosome, selection

## Abstract

Genome-wide patterns of diversity and selection are critical measures for understanding how evolution has shaped the genome. Yet, these population genomic estimates are available for only a limited number of model organisms. Here we focus on the population genomics of the pea aphid (*Acyrthosiphon pisum*). The pea aphid is an emerging model system that exhibits a range of intriguing biological traits not present in classic model systems. We performed low-coverage genome resequencing of 21 clonal pea aphid lines collected from alfalfa host plants in North America to characterize genome-wide patterns of diversity and selection. We observed an excess of low-frequency polymorphisms throughout coding and noncoding DNA, which we suggest is the result of a founding event and subsequent population expansion in North America. Most gene regions showed lower levels of Tajima’s D than synonymous sites, suggesting that the majority of the genome is not evolving neutrally but rather exhibits significant constraint. Furthermore, we used the pea aphid’s unique manner of X-chromosome inheritance to assign genomic scaffolds to either autosomes or the X chromosome. Comparing autosomal *vs.* X-linked sequence variation, we discovered that autosomal genes show an excess of low frequency variants indicating that purifying selection acts more efficiently on the X chromosome. Overall, our results provide a critical first step in characterizing the genetic diversity and evolutionary pressures on an aphid genome.

The pea aphid (*Acyrthosiphon pisum*) is an emerging genomic model system that exhibits traits that cannot be studied in most classic model organisms but are common in nature, such as polyphenisms, cyclical parthenogenesis, host−plant specialization, viral transmission, and bacterial symbioses (Brisson and Stern 2006; International Aphid Genomics Consortium 2010). The pea aphid genome, released in 2010 (International Aphid Genomics Consortium 2010), was the first for a hemimetabolous insect and revealed that the pea aphid genome harbors surprisingly high levels of gene duplications, exhibits lineage-specific gene losses, and shows evidence of metabolic coordination between bacterial symbionts and the aphid host (International Aphid Genomics Consortium 2010).

To date there has been no genome-wide analysis of genetic variation among pea aphid individuals, although studies examining a small number of loci have been performed (*e.g.*, Brisson *et al.* 2009; Ferrari *et al.* 2008; Simon *et al.* 2003). Studies in other organisms have shown that we can infer much about the processes that have shaped genome evolution by using sequence variation (Begun *et al.* 2007; Li and Stephan 2006). The emergence of next-generation sequencing technologies has enabled the investigation of sequence diversity from low genome coverage data for affordable prices. Here we report the first genome-wide analysis of genetic variation in the pea aphid using Illumina sequencing of genomic DNA (gDNA) at low coverage from 21 individuals collected from natural pea aphid populations. gDNA from each aphid clone was individually barcoded to enable us to assign polymorphisms to specific clones (*i.e.*, these were not pooled samples). We use these data to calculate levels of nucleotide diversity within the species and to assess selection acting on coding and noncoding DNA. We find an excess of low-frequency variants throughout coding and noncoding DNA, suggesting that the majority of the genome is acted upon by purifying selection. Our results achieve a critical step forward for the understanding of aphid biology, provide a valuable resource for those studying evolutionary and population genetic questions in this unique model system, and widen our understanding of genome-level population genetic patterns in insects.

Like most aphids, the pea aphid has a complex life cycle (Moran 1992), with unique hereditary patterns that are predicted to impact its genome-wide patterns of diversity. The pea aphid alternates between periods of asexual and sexual reproduction. In the spring, asexual females hatch from eggs that have overwintered. These females produce genetically identical daughters (barring spontaneous mutations) through a modified meiosis that lacks recombination and segregation of homologous chromosomes (Blackman 1987). Multiple generations of asexuality occur during the spring and summer months. In the fall, asexual females asexually produce sexual females and males. Both are genetically identical to their mother except that each male has randomly lost one X chromosome (Wilson *et al.* 1997). Both males and sexual females produce gametes with an X chromosome, which through fertilization produces an XX asexual female that hatches from an egg in the spring. Figure 1 follows the X chromosomes through the pea aphid life cycle.

**Figure 1:**
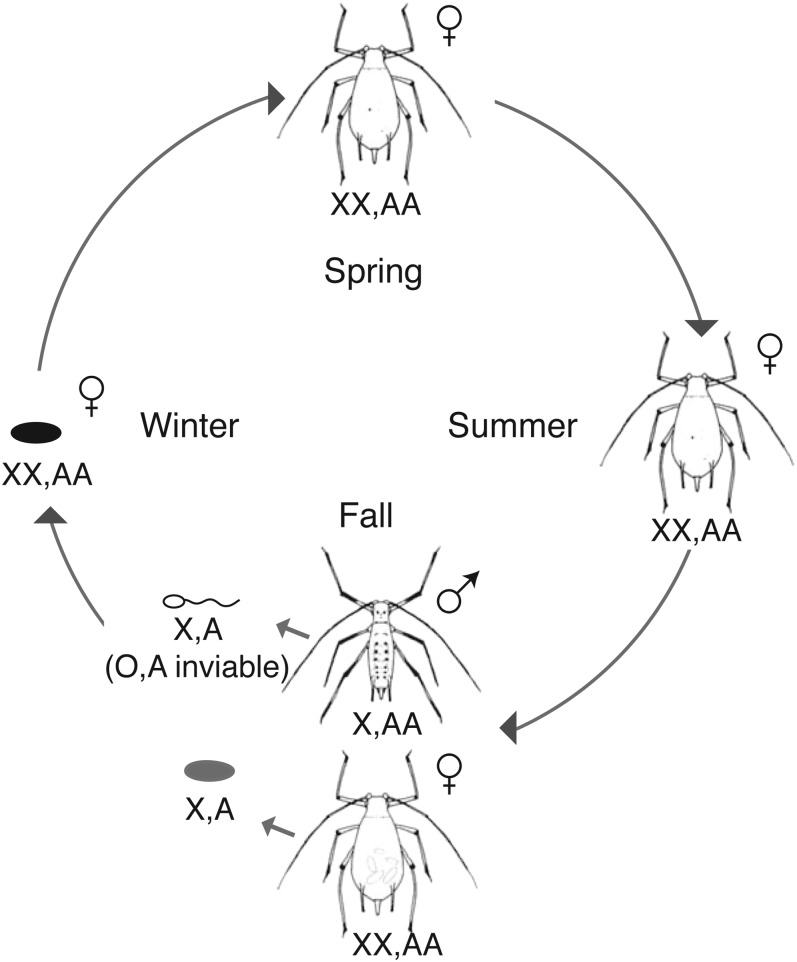
The pea aphid life cycle. In the spring, females hatch and then reproduce asexually throughout the summer. These females are diploid for all chromosomes (represented as XX, AA for the X chromosome and autosomes, respectively). In the fall, females asexually produce sexual females and males. Sexual females are genetically identical to the asexual females and the males are genetically identical except a male inherits only one of his mother’s X chromosomes (X, AA). The sexual females and males mate and the female lays fertilized eggs that overwinter. Only sperm with an X chromosome are viable (X, A), so all fertilized eggs are female (XX, AA).

We use our sequence data and a novel method to categorize pea aphid genomic scaffolds as X-linked or autosomal. To date, the pea aphid genome lacks a fine-scale linkage map. Without this resource, it has been impossible to assign individual genomic scaffolds to chromosomes. With our method, we assessed the probability that each scaffold is on the X chromosome *vs.* an autosome. The pea aphid’s unusual sex chromosome inheritance pattern means that each sex always contributes an X chromosome to the offspring of the sexual generation. In contrast, in well-studied XX/XO systems such as *Caenorhabditis elegans*, the female contributes an X chromosome 100% of the time and the male 50% of the time. In these latter systems, X-linked *vs.* autosomal genes can be differentially affected at the sequence level by sex-specific reproductive success, sex-biased mutation rates, sex-biased dispersal, demographic processes, and recombination rates (reviewed in Ellegren 2009). In pea aphids, both the female and the male always pass an X chromosome to the next generation. This should result, theoretically, in both the X chromosome and the autosomes being affected equally by these processes (Jaquiery *et al.* 2012). Indeed, simulations demonstrate that the pea aphid’s unique X-chromosome transmission results in similar effective population sizes and predicted levels of genetic diversity for X chromosomes and autosomes under neutral evolution (Jaquiery *et al.* 2012). Here we test these predictions on a genome-wide scale using our predicted X-linked *vs.* autosomal genes. In conflict with theoretical predictions, we find an excess of low frequency variants on autosomal genes relative to X-linked genes. This pattern may be due to a number of factors (differences in recombination rates, gene density, gene classes present, etc.) that potentially differ between the pea aphid autosomes and X chromosome. We suggest that this pattern may be largely explained by the higher efficiency of purifying selection on the X chromosome because of recessive lethal mutations in the males.

## Materials and Methods

### Aphid lines

We collected pea aphids from alfalfa fields in California, Massachusetts, and New York. Clonal lineages, also known as lines, were genotyped at up to 12 restriction fragment length polymorphic (RFLP) loci to verify that they were genetically unique. Aphid lines were transferred to an incubator that mimicked fall photoperiod (13:11-hr light/dark cycle) until they produced males. All lines selected for analysis produced both winged and wingless males due to heterozygosity at the male wing determination locus, *aphicarus* (Braendle *et al.* 2005; Caillaud *et al.* 2002). For each line, we collected from four to ten males of each phenotype, winged or wingless. Because males are produced asexually with no recombination, all males produced by a line within a phenotypic class (winged or unwinged) are genetically identical barring spontaneous mutations. We used a total of 21 unique lines (7 from New York, 4 from Massachusetts, and 10 from California), with winged and wingless males from each line for a total of 42 samples. After sequencing, data from males of the same line were combined such that we had diploid data from 21 lines for subsequent analysis.

### Sample preparation and sequencing

We prepared samples for sequencing on an Illumina Genome Analyzer as follows. For each male sample, we isolated gDNA, sheared the gDNA by sonication, and selected 300- to 500-bp sized pieces on a 2% agarose gel. We then ligated an adapter sequence containing a three-base barcode and used 15 rounds of amplification using Phusion Taq polymerase (New England Biolabs). Four groups of 10 males with unique bar codes were combined in equal quantities after being quantified on a Qubit fluorometer (Invitrogen). An additional sample was made by combining the two remaining barcoded males. Samples were sequenced on an Illumina Genome Analyzer by the use of 15 lanes of 54nt sequencing. After initial analysis of these data, we determined that samples were not being sequenced in equal quantities. Insufficient amplified material remained, so we returned to the original libraries for each underrepresented male and amplified them individually by polymerase chain reaction. We combined them in equal quantities and sequenced each of four libraries again, two of them with 54nt Illumina sequencing and two of them with 36nt sequencing. The additional sequence data were combined with the original data before filtering and analyses. An additional line (the F1 line of Braendle *et al.* 2005) was independently sequenced using 100-bp, paired-end Illumina sequencing. One lane was run for the winged males and one lane for the wingless males. Details of coverage for each line can be found in Supporting Information, Table S1.

Winged and wingless males were sequenced separately for each line for use in association mapping of the *api* locus, which is not the focus here. We therefore combined the wingless and winged male data to produce a single sequence data set for each line.

### Sequence alignment and filtering

Version 2.1 of the pea aphid genome was downloaded from ftp://ftp.ncbi.nih.gov/genomes/Acyrthosiphon_pisum/aps_ref_Acyr_2.1_chrUn.fa.gz. The mitochondrial genome was not used. We aligned sequence reads to the genome using the Burrows-Wheeler Aligner version 0.6.1 using default settings (Li and Durbin 2010), and alignment pileup format files were generated using Sequence Alignment/Map Tools (SAMtools) version 0.1.17 (Li and Durbin 2009). For each of the 21 lines, we determined the consensus sequence using the following criteria: only bases with a quality score >20 (99% base call accuracy) were considered. If >80% of the bases at a position were the same, this base was used as the consensus base. If <80% were the same, the base was marked as an ambiguous base (N); approximately 0.001 bases fit into this category (overall = 0.00105, autosomes = 0.00103, X chromosome = 0.00113). These consensus sequences for each line were used for downstream population genetic analyses.

### Identification of X-linked scaffolds

We sequenced winged and wingless males generated from the F1 clonal line (Braendle *et al.* 2005) at high coverage. These two male genotypes have the exact same autosomes but differ in which X chromosome they carry. Thus, their genome sequences should only differ for scaffolds that are located on the X chromosome. Using every genomic position that had a base pair call for both winged and wingless F1 males, we calculated the pairwise difference per base pair between them. Scaffolds on the X chromosomes should have many pairwise differences, whereas scaffolds on autosomes should have no differences.

Multiple factors could affect the pairwise difference value. X-chromosome scaffolds with high diversity should have high pairwise differences, and thus should be easier to identify than X-chromosome scaffolds with low diversity. Both autosomal and X scaffolds may contain errors that appear to be pairwise differences. We have used strict cutoffs to minimize these occurrences (see *Sequence Alignment and Filtering* section above). Furthermore, autosomal scaffolds may erroneously have differences between the winged and wingless male if a heterozygous base has unequal and opposite read coverage of their two alleles, such that one male has only has reads from one allele and the other male from the other allele. This would appear as a pairwise difference. These issues make it particularly difficult to differentiate low diversity X-linked scaffolds from autosomal scaffolds with high diversity.

We reasoned that if we corrected by the amount of diversity on a scaffold we should be able to better differentiate X-linked from autosomal scaffolds. X scaffolds with low diversity will have low pairwise differences. Autosomes with high diversity are likely to have some heterozygous bases that appear to be pairwise differences, but at a much lower rate than the overall diversity. These two hypothetical scaffolds would have a similar number of pairwise differences per base pair, but different pairwise differences when corrected for diversity levels. We therefore created a corrected differences calculation as follows:

Corrected differences=Pairwise differences between males−π

π (Nei and Tajima 1981) is an estimator of genetic diversity, calculated as the average pairwise differences per bp within a population. We independently calculated π from our pool of 21 lines. When we calculated π, each line included sequence data from both winged and wingless males and thus included both autosomal and X-linked data (both X chromosomes).

For any scaffold that is on the X chromosome, if the scaffolds observed have a similar number of pairwise differences/bp as the average pairwise differences/bp of the whole population, then the corrected difference would be 0. If a scaffold is located on an autosome, we expect the number of pairwise differences observed to be much lower than π, creating a negative score. This correction also creates three advantageous characteristics: we should have two normally distributed populations of scaffolds, we have a predicted mean of zero for the smaller population of scaffolds (the X chromosome scaffolds), and we should be able to better differentiate X scaffolds with low diversity from autosomal scaffolds with high diversity.

The resulting values fall into two overlapping populations of scaffolds, one centered at 0 and the larger population centered at a negative value, presumably representing scaffolds on the X chromosome and the autosomes, respectively. We found that a Student’s *t* distribution best fit our data. We used maximum likelihood to estimate the mean and variance of the two populations. We used these data to assign a probability score for each scaffold as belonging to the X-chromosome group or the autosomal group.

### Population genetics calculations

The sequence data were used to generate Fasta files for all genomic scaffolds >100 kb for each aphid line. For each gene the pea aphid v2.1 annotation was used to categorize the following regions: whole gene (coding, UTRs, and introns), gene plus 5 kb upstream and downstream (the gene plus likely regulatory DNA), Coding sequence (CDS), 5′ untranslated regions (UTRs), 3′ UTRs, introns, 10 kb upstream, and 10 kb downstream. Population genetic statistics were generated using the *compute* program included with the libsequence library (Hudson *et al.* 1992; Thornton 2003). Only sequence sets with at least 50 polymorphisms were included in the analysis for any scaffold, gene, or gene region (CDS, UTRs, introns, upstream, and downstream).

### Scaffold location verification

Genomic DNA from females, winged males, and wingless males of the F1 line was extracted using the DNeasy Blood & Tissue Kit (QIAGEN). For each scaffold to be verified, polymerase chain reaction primers were designed to amplify a 100- to 300-bp DNA fragment containing an RFLP in the females (primers and restriction enzyme are listed in Table S2). The fragment was amplified from females and each male morph, cut with the relevant restriction enzyme overnight, and run on a 4% agarose gel. Autosomal loci show the pattern of heterozygosity in the females as well as each male because females and males are diploid for all autosomes. X-linked loci show the pattern of female heterozygosity and male homozygosity because males are haploid for the X.

### Gene ontology analysis

The program Blast2GO was used to blast and annotate the genes identified in pea aphid annotation version 2.1 using default parameters (Conesa *et al.* 2005). Fisher exact tests were performed to determine enriched Gene Ontology (GO) terms in gene lists using a false discovery rate (FDR) <0.05.

## Results and Discussion

We sequenced 21 genetically distinct lines of pea aphids using the Illumina platform. In all, we generated more than 278 million sequencing reads, which resulted in an average coverage of 5.7 individuals sequenced per site (of sites with any sequence information) across the genome. Some lines provided more sequence coverage than others, with the relative contribution of each noted in Table S1. We sequenced the F1 line to high coverage for the purpose of identifying X-linked *vs.* autosomal genomic scaffolds (15% of the 5.7 bp average total at each site). All other lines account for between 2% and 6% of the 5.7 bp average total at each site.

### Aphid samples from California *vs.* the Northeast show little population differentiation

Pea aphids are native to Eurasia but have been introduced to North America multiple times over the last 150−200 years (Blackman and Eastop 2000; Davis 1915; Sanderson 1900). They feed on members of the pea family and are found on field crops such as alfalfa (genus *Medicago*) and clover (genus *Trifolium*), which are distributed across the United States. During the asexual portion of the pea aphid life cycle, females are wingless or winged. Wingless morphs are largely immobile, whereas winged morphs can disperse from field to field or even travel large distances via passive migratory flights in upper air currents, traveling as far as 1000 km in a single flight (Robert 1987). The winged morph’s potential for eventual gene flow may or may not be sufficient to eliminate population subdivision in the United States. To test this, we calculated F_st_ levels across the genome, comparing 11 pea aphid lines from the Northeast US (New York and Massachusetts) and 10 from California. We observed no structure, with an overall F_st_ value of -0.021. We conclude that pea aphid populations in the United States function as a single, panmictic population.

Pea aphids in the northeast demonstrate strong host plant specialization (Via 1999; Via *et al.* 2000), whereas in California both specialists and generalists exist (Leonardo and Muiru 2003). All pea aphids used in this study were collected from alfalfa, so our conclusions apply specifically to pea aphids that feed on this host plant. F_st_ values for comparisons of clover and alfalfa specialists in France were previously found to be around 0.1 (allozymes, F_st_ = 0.098; microsatellites, F_st_ = 0.104) (Simon *et al.* 2003), indicating that, as expected, genetic differences between host plant specialists are much greater than genetic differences within a host plant specialist.

Despite the negligible overall population subdivision observed here, individual genes exhibited high F_st_ values. Specifically, 164 genes of the total 36,961 annotated genes (annotation version 2.1) had an F_st_ value greater than or equal to 0.5, indicating strong differentiation between the northeast and California populations. All genes and their F_st_ values can be found in Table S3. One possible explanation for this strong differentiation is selection. The 164 highly differentiated genes comprised significant (FDR < 0.05) GO categories related to localization and transport; oxidation reduction and carbohydrate catabolic processes; and DNA mismatch repair. Pea aphids on the east and west coast of the United States face different challenges from their host plants. Alfalfa in California develops under hot, dry conditions whereas alfalfa in the northeastern United States experiences cooler and wetter climates and a shorter growing season. If selection is responsible for these genes having high F_st_ values, it may be because pea aphids on the east and west coast of the United States experience differential selection related to the tendency to disperse, survival during dispersal, or subsequent adaptation to different metabolic products from their host plants.

Alternatively, genes with high F_st_ values might be involved in reproductive mode divergence. The pea aphid shows genetic variation for whether and when it alternates between sexual and asexual reproduction or whether it remains parthenogenetic (Frantz *et al.* 2006). Pea aphid populations in the northeastern United States must undergo a sexual generation in the fall to produce eggs that can experience diapause through the cold temperatures that cause adult mortality. In contrast, asexual reproduction is potentially more advantageous year-round in the much warmer climate of California. Under any hypothesized scenario for local adaptation, the genes with high F_st_ values are excellent candidates for future studies verifying whether they are indeed under selection. The majority of the genome shows no differentiation between the northeastern United States and California aphids. Because there is no systematic differentiation, we treated our samples as a single population for further analyses.

### Diversity and selection across the pea aphid genome

We documented levels of genetic diversity across the genome using two estimators of genetic diversity: Watterson’s θ (θ^w^) and π. θ^w^ estimates genetic diversity as a function of the number of segregating sites (Watterson 1975), whereas Nei and Tajima’s π uses the number of nucleotide differences per pair of sequences (Nei and Tajima 1981). Thus, θ^w^ gives equal weight to all polymorphisms, whereas π is more strongly impacted by frequent polymorphisms. θ^w^ and π for all sites across the genome were 0.0050 and 0.0045, respectively.

We then looked at different potentially functional regions within each gene in the pea aphid genome to determine whether they exhibited markedly different levels of nucleotide diversity. Specifically, we calculated diversity measures for coding regions (CDS) and for the following noncoding regions: 5′ UTRs, 3′ UTRs, introns, the 10-kb upstream intergenic region (IGR), and 10-kb downstream IGR. The coding region is usually one of the most constrained regions in the genome. 5′ and 3′ UTRs are often implicated in posttranscriptional regulation, introns often impact translation and can potentially contain *cis*-regulatory elements, and IGRs can contain regulatory elements that affect transcription and chromatin state (Bernstein *et al.* 2012). Values of θ^w^ and π for each region are listed in Table 1. The UTRs display the highest levels of diversity, followed by introns and IGRs. The CDS had the lowest diversity suggesting that, unsurprisingly, the coding region is strongly constrained, whereas noncoding regions are not.

**Table 1:** Coverage and measures of polymorphism in coding and noncoding DNA of the pea aphid

	Coverage*^a^*	θ^w^	π	Tajima’s D
Gene				
Overall	6.07	0.00476	0.00408	−0.83724
Autosomal	6.19	0.00477	0.00413	−0.82363
X	4.91	0.00459	0.00407	−0.72507
CDS				
Overall	7.96	0.00432	0.00353	−0.86371
Autosomal	8.12	0.00424	0.00348	−0.86380
X	6.47	0.00432	0.00366	−0.75407
5′ UTR				
Overall	7.73	0.00491	0.00415	−0.65300
Autosomal	7.88	0.00494	0.00418	−0.64950
X	6.34	0.00470	0.00406	−0.58506
3′ UTR				
Overall	4.62	0.00444	0.00395	−0.49067
Autosomal	4.70	0.00440	0.00394	−0.48398
X	3.83	0.00415	0.00379	−0.39306
Introns				
Overall	5.43	0.00478	0.00418	−0.74401
Autosomal	5.55	0.00483	0.00425	−0.74748
X	4.31	0.00482	0.00441	−0.57408
Upstream IGR				
Overall	5.35	0.00502	0.00441	−0.82091
Autosomal	5.47	0.00504	0.00444	−0.82898
X	4.25	0.00481	0.00434	−0.72661
Downstream IGR				
Overall	5.39	0.00503	0.00442	−0.82255
Autosomal	5.52	0.00508	0.00447	−0.83013
X	4.22	0.00486	0.00438	−0.71935

Only 29178 of 36990 genes were assigned to autosomes or the X. CDS, coding regions; UTR, untranslated region; IGR, intergenic region.

a Coverage is measured as the average number of lines represented per base pair.

**Figure 2:**
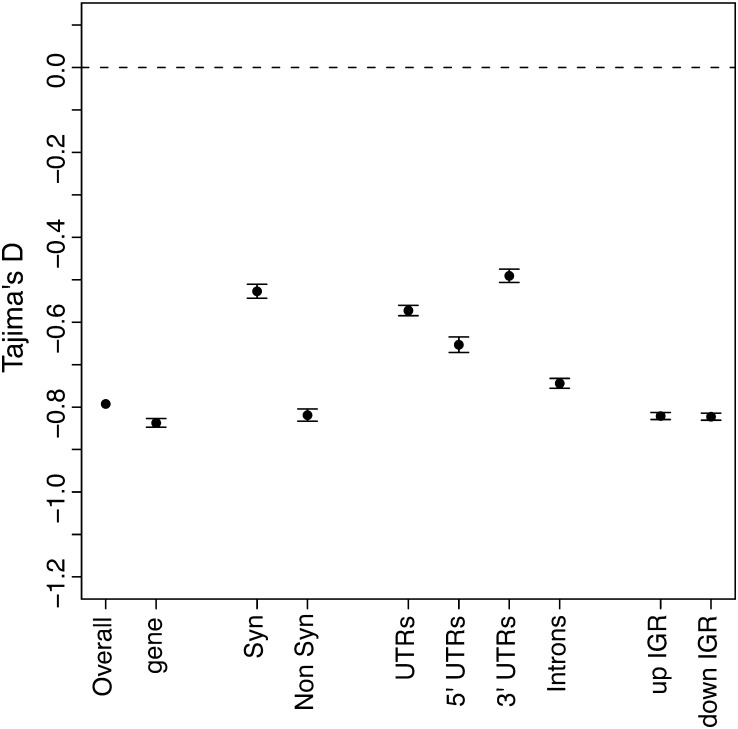
Mean Tajima’s D values for coding and noncoding DNA. The mean Tajima’s D values are given for different loci with bars indicating four SEs. The dotted line represents the neutral expectation. The labels are gene (exon, UTRs and introns), Syn (synonymous coding), Non Syn (non-synonymous coding), UTRs (untranslated regions), Introns, and up IGR (10 kb upstream intergenic region) and down IGR (10 kb downstream intergenic region).

Tajima’s D test compares θ^w^ and π to identify sequences that are not evolving neutrally (Tajima 1989). If a region is evolving neutrally, the two diversity measures should be equal (D = 0), whereas selection and demographic factors cause these two estimators to differ (D ≠ 0) (Tajima 1983). We calculated Tajima’s D for each gene region to determine which regions are under selection (Figure 2). Typically synonymous sites are considered neutral and thus would be expected to have a Tajima’s D value of 0. Interestingly, we observed a negative (−0.53) Tajima’s D value for synonymous sites. The low Tajima’s D may be due to the rapid range expansion of the pea aphid in North America since it was introduced approximately 150−200 years ago. This expansion would systematically affect all regions of the genome, causing lower D values (Tajima 1989). Also, sequencing errors can skew θ^w^ estimates toward larger values, which can in turn result in more negative Tajima’s D values (Achaz 2008). Although we conservatively filtered out poor base pair calls (see *Materials and Methods*), the low sequencing coverage has likely impacted our results. Therefore, the absolute Tajima’s D values may be impacted by both demographic effects and sequencing errors. We expect these factors to affect each gene region equally. Thus, differences between regions are likely to be the result of difference in relative selection.

Synonymous site substitutions typically most closely meet the neutral expectation, so we compared nonsynonymous sites to synonymous sites. As expected, nonsynonymous sites exhibited lower Tajima’s D values, indicating selection (*P* < 0.001, Mann-Whitney *U*-test). Additionally, introns, 5′ UTRs and the 10-kb IGR on either side of each gene also showed evidence of purifying selection, with all gene regions except the 3′ UTRs having significantly (*P* < 0.001, Mann-Whitney *U*-test) lower Tajima’s D values than synonymous sites (Figure 2). *Arabidopsis thaliana* (DeRose-Wilson and Gaut 2007) and *Drosophila melanogaster* (Andolfatto 2005) similarly show that most noncoding regions exhibit evidence of purifying selection. However, unlike *Drosophila*, where UTRs showed the strongest selection of the noncoding regions, pea aphid UTRs exhibit the least constraint of the noncoding DNAs. Upstream and downstream IGR exhibit similar Tajima’s D values as nonsynonymous sites.

To compare individual genes, we examined D values for the entire gene region (UTRs, coding, and introns) and tested whether particular gene functional classes were significantly overrepresented in the lowest or highest 10% of Tajima’s D values. GO terms enriched for the lowest 10% Tajima’s D values (a range of −2.41 to −1.48) relate to DNA binding, including RNA-directed DNA polymerase activity and double-stranded DNA binding (FDR < 0.05). GO terms enriched for the greatest 10% Tajima’s D values (a range of −0.24 to 1.53) mainly involve metabolic functions, such as carbohydrate metabolic process and peptide transporter activity. A full list of GO terms, with their associated genes, is available in Table S4. The overrepresented GO terms describing the genes in the top 10% of Tajima’s D values mirror the terms that were significantly enriched for genes highly differentiated between Northeast and California samples (F_st_ values >0.5). Almost half (71 of 164 genes) of the highly differentiated genes were also represented in the top 10% of Tajima’s D values. This may be partially explained by our sampling equally from California and the Northeast, but it is also possible that there is population structure or balancing selection at these loci.

### Discovery of X-linked *vs.* autosomal genomic scaffolds and genes

One of the challenges of many genome assemblies, particularly in nonmodel organisms that do not have detailed physical or linkage maps, is the large number of scaffolds that are generated and cannot be accurately assigned to a particular chromosome. In the case of the pea aphid, 23,924 scaffolds exist and it is unknown on which of the four chromosomes (one X, three autosomes) they belong. We developed a unique method to identify scaffolds on the X chromosome that takes advantage of the aphid’s method of male production during the life cycle (Figure 1), in which males are genetically identical to their mother except that they inherit only one of her two X chromosomes (females are XX and males XO). We used high coverage genome sequence information from males produced from a single genetic clone (F1 line) heterozygous for the *aphicarus* (*api*) locus (Caillaud *et al.* 2002), an X-linked locus that controls a male wing polymorphism in the species. Because males are produced by the loss of a single X chromosome in the mother, they will inherit either the winged or wingless allele of *api*. There is no recombination on the X chromosome when asexual females produce males. Therefore, the wing phenotype is a marker for which X chromosome the male inherits from his mother and the *api* locus is in complete linkage with the remainder of the X chromosome. These males contain an identical set of autosomes to their mother and to each other.

We compared the genome sequences between winged and wingless brothers of this F1 line to categorize scaffolds as located on the X or autosomes. If a scaffold is located on the X chromosome, the winged and wingless males should have sequence differences due to polymorphisms on the two different X chromosomes. A scaffold located on an autosome should be identical in the winged and wingless male because they inherit the same set of autosomes from their mother. However, in practice, sequence data are not perfect. Sequencing errors can result in base positions that appear to have differences but those differences are not truly present in their genomes. Furthermore, on autosomes, some heterozygous positions may have reads from only one particular allele in one male and only reads from the other allele in the other male, again appearing as a difference. Thus a single informative site does not conclusively show whether a scaffold is X-linked or autosomal. But when we examine a large number of sites, we predict that scaffolds from the X and autosomes will have different patterns.

**Figure 3:**
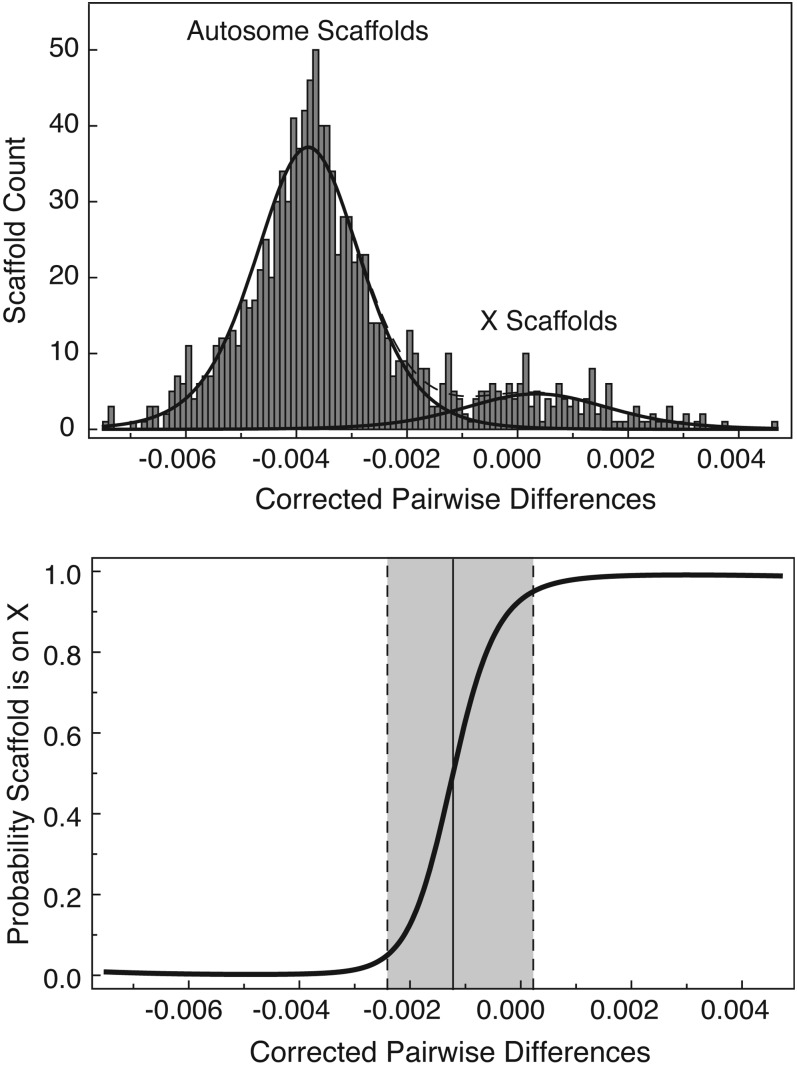
The distribution of the corrected pairwise difference and the associated probability that a scaffold is on the X chromosome. The top panel shows the frequency of scaffolds with a given corrected pairwise difference. The lines represent the fitted models. The bottom panel shows the probability of a scaffold being on the X chromosome as a function of the corrected pairwise difference. The dotted lines indicate the 0.05 and 0.95 probability values and the solid line represents the 0.5 probability value.

We examined the largest 1106 genomic scaffolds, each >100 kb in length, which together contain >85% of the genome (461,823,477 bp of the 541,675,471 bp total length). We used this cutoff to have sufficient sequence information for assessing pairwise sequence differences on any given scaffold. We produced a difference score for each scaffold that included the number of sites that differed between winged and wingless males. X scaffolds should have many more differences than autosomal scaffolds. X scaffolds with a large number of polymorphisms should have a larger difference score than X scaffolds with a small number of polymorphisms, and thus are easier to identify as belonging on the X. Unfortunately, autosomal scaffolds with a large number of polymorphisms will have a greater incidence of sequencing the opposite allele from each male than autosomes with a lower number. In some cases, the extremely polymorphic autosomes may have a similar number of differences as X chromosomes with very low levels of polymorphism. Thus, we corrected this score by subtracting the diversity on the scaffold (π, as calculated from all 21 lines) from the number of differences (see *Materials and Methods* for details). We expected to observe a bimodal frequency distribution of corrected difference scores by scaffold, with negative scores corresponding to autosomal scaffolds (many more polymorphisms than differences between brothers) and scores with a mean of zero corresponding to X-linked scaffolds (similar number of differences between brothers as the average polymorphism on the scaffold). The X chromosome is only one of the four total chromosomes comprising the 540 Mb genome, so we further expected that the autosomal scaffolds would be a much larger percentage of the total scaffolds as compared to the X-linked scaffolds. As predicted, the scaffolds fell into two peaks with autosomal scaffolds in larger numbers than X-linked scaffolds (Figure 3).

The two distributions overlapped slightly, so we used maximum likelihood to model the distribution to obtain a probability that a given scaffold is on the X. The probability values and how they correspond to the corrected differences per scaffold is visualized in Figure 3. Table S5 lists the scaffold names and their probability of being on the X.

For further analysis we assigned scaffolds with an “X probability” of <0.5 as autosomal and a probability >0.5 as being X-linked. This resulted in 952 autosomal scaffolds and 154 X-linked scaffolds. These scaffolds housed 26,250 and 2928 genes, respectively.

In males, we expect autosomal DNA to be sequenced twice as often as X DNA because they have a 2:1 ratio in the genome. Predicted autosomal scaffolds had, on average, 18.06 reads per bp, whereas predicted X scaffolds had 10.33, a ratio of 1.74:1. This is consistent with a large number of the scaffolds being correctly identified. The ratio was not exactly 2:1, suggesting some scaffolds may have been misidentified (see below for one explanation).

**Figure 4:**
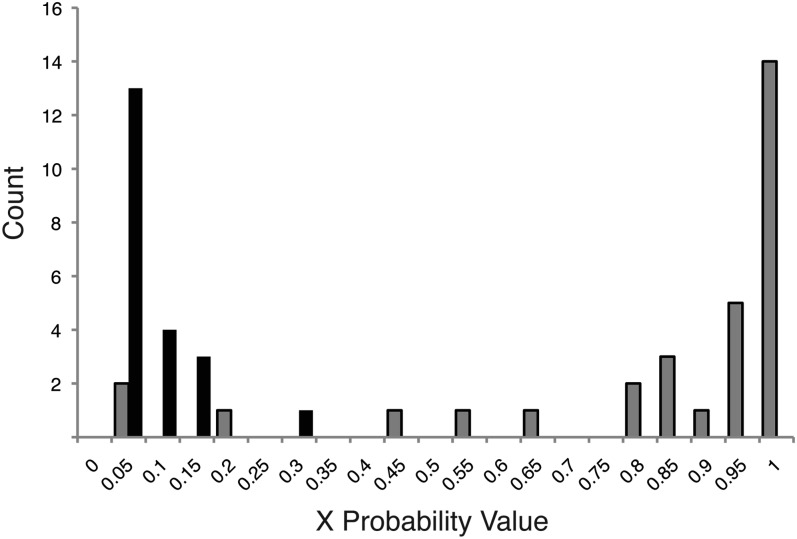
The X probability values associated with scaffolds verified via RFLP analysis as X-linked (gray bars) or autosomal (black bars). Counts for the numbers of scaffolds that fall within each X probability value category are shown on the y-axis.

To verify our method of assigning scaffolds to X or autosomal chromosomes, we independently identified 31 scaffolds on the X and 21 scaffolds on autosomes using RFLP analysis on male *vs.* female pea aphids (see *Materials and Methods*). All 27 scaffolds with an X probability >0.5 were verified as X-linked. 21 of 25 scaffolds with an X probability <0.5 were verified as autosomal, whereas the remaining four were verified as X-linked. The verified scaffolds and their X probabilities are illustrated in Figure 4 and the RFLP primers for each are listed in Table S2.

We observed that miscategorization was most likely among larger scaffolds. We discovered that three scaffolds we examined (not included in the above numbers) were chimeras, a misassembled scaffold containing both X-linked and autosomal DNA. For a chimeric scaffold, an RFLP on one side of the scaffold demonstrated it to be X-linked and an RFLP on the other side demonstrated it as autosomal. These scaffolds have X probabilities of 0.03, 0.13, and 0.83. Based on this observation, we hypothesize that some of the larger scaffolds are chimeras of both autosomal and X-linked DNA incorrectly merged during genome assembly because the larger the scaffold, the greater the probability that an assembly error occurred. Depending on the levels of polymorphism within a given scaffold, it could skew a primarily X-linked chimera toward an autosomal probability score or vice versa. Furthermore, our verification method was based on a single SNP and therefore would verify the scaffold as X-linked or autosomal based on that position, when in fact the scaffold was a mixture of the two DNAs. We conclude that our assignment method works well, but that the genome annotation may have a large number of chimeric scaffolds. Further, we conclude that there is high confidence in our method when it assigns a high X-linked probability value since the scaffolds with high probability values have been verified as truly X-linked. However, a small percentage of X-linked scaffolds or chimeras between X-linked and autosomal scaffolds will be present among the scaffolds identified as autosomal.

The X chromosome has previously been estimated to be 30% of the pea aphid genome based on chromosome photographs (Bizzaro *et al.* 2000; Braendle *et al.* 2005; Mandrioli *et al.* 1999). With our data, we can now take a more detailed look at the ratio of X-linked to autosomal DNA. The total length of the combined scaffolds examined here was 461 Mb. The total length of the assembled genome is 540 Mb. The difference in these two genome sizes is because we restricted our analyses to scaffolds larger than 100 kb to have enough data to estimate corrected differences between winged and wingless males. From the scaffolds we examined, the X-linked scaffolds account for 11.7% of the DNA (53,889 kb) and the autosomal scaffolds account for 88.3% of the DNA (407,934 kb), in total harboring 29,178 of the 36,961 genes in the current (version 2.1) pea aphid annotation. We have therefore assigned the majority of the annotated genes to putative X or autosomal locations. Our estimate of 11.7% of the genome being X-linked is different than the 30% previously estimated from visual inspection of chromosome preparations. A large portion of the pea aphid X chromosome appears to be heterochromatic (Mandrioli and Borsatti 2007). This highly repetitive DNA would not be present in assembled scaffolds, which are primarily euchromatic DNA. Furthermore, some of this discrepancy is likely due to the scaffolds we used or did not use for our analysis. In particular, the large amount of heterochromatic DNA (Mandrioli and Borsatti 2007) can lead to genome assembly problems, resulting in small genomic scaffold sizes. X chromosome sequences are thus likely over-represented in the smaller scaffolds and therefore underrepresented in the scaffolds we used here. Finally, chimeric scaffolds assigned to autosomes could include large portions of X chromosomal sequence, particularly among the larger scaffolds. These would not be included in the final X-linked DNA count considered here.

We find that the autosomes have greater gene density than the X chromosome (on average a gene every 15,540 bp on autosomal scaffolds and 18,405 bp on X-linked scaffolds). Furthermore, the average gene length is longer on the autosomes (10,203 bp *vs.* 9,549 bp). Neither genes on the X chromosome nor genes on the autosomes were significantly enriched for any gene ontology categories after multiple comparison correction.

### Sequence diversity differs between the X and autosomes

In most XX/XY organisms, genetic diversity is expected to be higher on autosomes than the X chromosome because of the higher effective population size of the autosomes (reviewed in Ellegren 2009). The pea aphid, with its XX/XO sex determination, presents a different scenario: the effective population sizes of the X chromosome and autosomes are equivalent because a mating female and male each contribute an X chromosome to the next generation (Jaquiery *et al.* 2012) and thus there is no *a priori* expectation of lower X diversity. The neutral expectation, therefore, is equal levels of genetic diversity between X chromosomes and autosomes. A previous study in pea aphids found similar genetic diversity on the X compared with autosomes using microsatellite repeats (Jaquiery *et al.* 2012). Here we examine the relative genetic diversity on a genome-wide scale. We calculated levels of θ^w^ and π for each scaffold and compared autosomal and X-linked chromosomes. We found that autosomal scaffolds had slightly greater θ^w^ (0.005028 *vs.* 0.004833, ratio of 1.04) and π values (0.004459 *vs.* 0.004414, ratio of 1.01) compared with X-linked scaffolds. To determine whether these differences were significant, we compared the θ^w^ and π values for genes (exons plus introns and 10 kb of intergenic regions on each side of the gene) on the autosomes *vs.* X chromosome. For both θ^w^ and π, the autosomal genes had significantly greater values than the X chromosome genes (Mann-Whitney *U*-test, *P* < 0.001). The same results were obtained whether we considered X-linked genes as those with an X-probability value of greater than 0.5 and autosomal as less than 0.5 or if we considered the much more stringent criteria of X-linked genes as those with an X-probability value of greater than 0.95 and autosomal as less than 0.05. We conclude that autosomal DNA harbors slightly greater nucleotide diversity than X-linked DNA despite the 1:1 X:A diversity expectation, although the differences are subtle.

**Figure 5:**
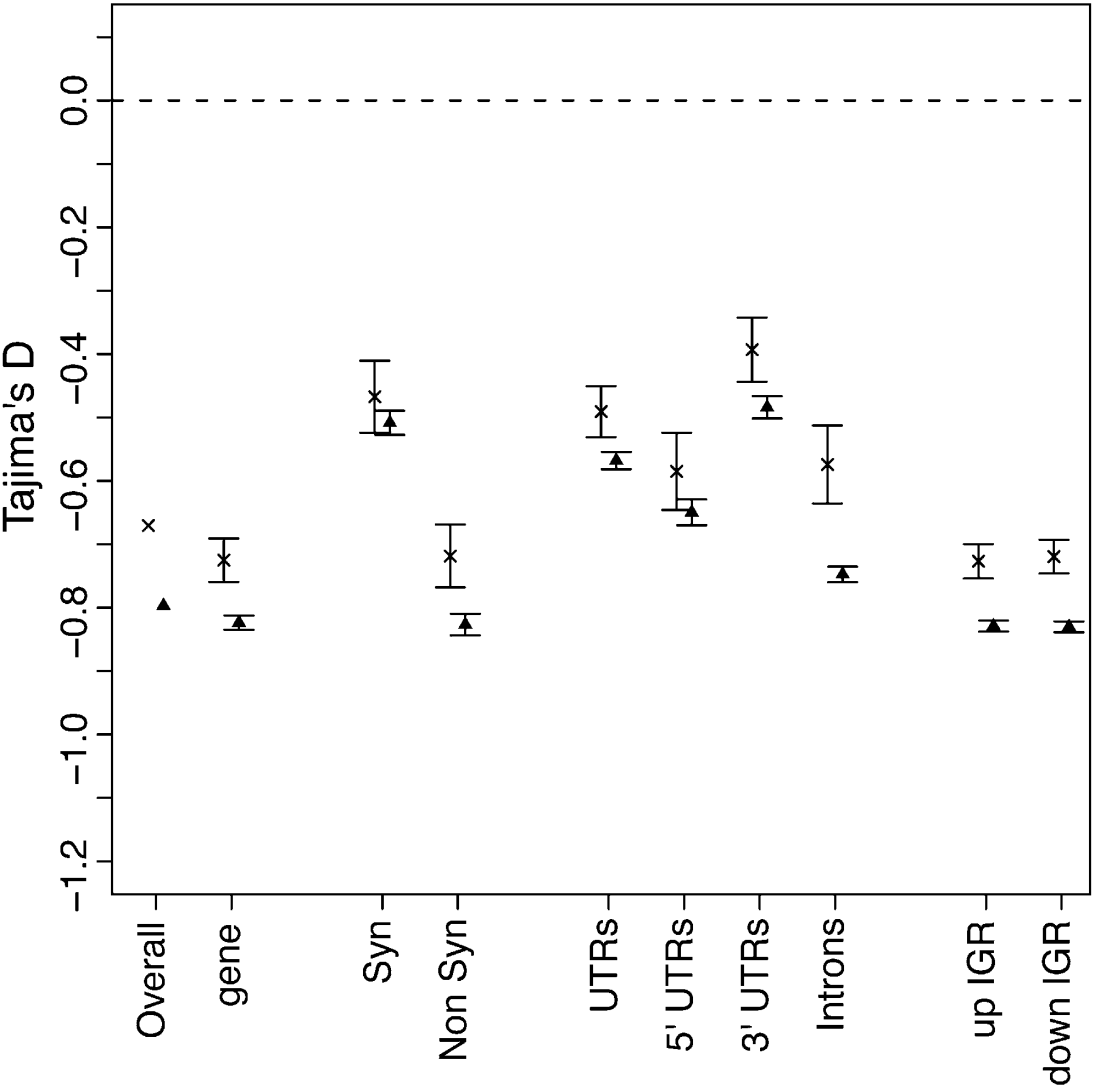
Mean Tajima’s D values for autosomal and X-linked scaffolds. The mean values are given for genes or gene parts with bars indicating four SEs. The “x” symbols indicate the mean values of scaffolds predicted to be on the X chromosome and the triangle symbols indicate the mean values of scaffolds predicted to be on autosomes. The dotted line represents the neutral expectation.

Deviations from the 1:1 X:A diversity expectation could be due to the action of natural selection. We therefore investigated whether selection affects X *vs.* autosomal DNA sequences differentially, again using Tajima’s D as an indicator of selection. We examined whole genomic scaffolds, as well as genes and parts of genes. In all cases, Tajima’s D values were negative and significantly lower for autosomal compared to X-linked genes (*P* < 0.001, Figure 5; the same result was obtained with the more stringent criteria). We anticipated that synonymous sites would show similar values of Tajima’s D regardless of their location, again because they are thought to evolve at a nearly neutral rate. Consistent with this expectation, synonymous differences show the smallest difference between X and autosomes (Figure 5). In all other comparisons of genes or gene parts, autosomal genes exhibited a larger signal of purifying selection.

We compared the Tajima’s D values of the 52 scaffolds that we had independently verified via RFLP analysis as autosomal or X-linked. The results from this subset of scaffolds were very similar to what we detected from the total data set. We observed an average Tajima’s D of -0.64 for X-linked scaffolds and −0.78 for autosomal scaffolds. Further, the differences between them were highly significant (Mann-Whitney *U*-test, *P* = 2 × 10^−5^). However, the marginally greater θ^w^ and π differences we observed for autosomal relative to X-linked genes across all scaffolds was not supported by polymorphism measure comparisons within these 52 scaffolds (Mann-Whitney *U*-test, *P* = 0.93 and 0.40 for θ^w^ and π, respectively).

In summary, autosomal genes show marginally higher levels of nucleotide diversity and an excess of low-frequency variants. What could account for the greater number of low-frequency polymorphisms on the autosomes relative to the X chromosome? Recall that pea aphid females have two X chromosomes whereas males have only one. Although the autosomes and the X chromosome have the same effective population size (Jaquiery *et al.* 2012), their copy number difference means they respond to selection differently. Recessive X-linked alleles will be exposed to selection every time they are present in males because no other allele is present. Therefore, low-frequency recessive detrimental mutations, which would rarely be homozygous on an autosome, would frequently be under selection if on the X chromosome (Vicoso and Charlesworth 2006). Selection can fix beneficial and remove deleterious recessive mutations more effectively on the X (Charlesworth *et al.* 1987; Rice 1984). However, because of this effectiveness, selection can remove or fix alleles on the X before they can affect the frequency spectrum of the surrounding polymorphisms in the whole population. Thus, even though selection acts more effectively on the X chromosome, that selection may not have a large effect on the frequency spectrum of the entire region. We suggest that this could account for the pattern we observe here.

Interestingly, there is evidence that the pea aphid X chromosome is evolving faster than the autosomes. Jaquiery *et al.* (2012) found marginally significant (*P* = 0.03) acceleration of evolution of X relative to autosomal genes as measured by dN/dS in two of three comparisons of pea aphid genes to other aphid species for several hundred genes. Consistent with this, we find that pea aphid genes on the X chromosome are less likely to have potential orthologs (hits to the “nr” database via Blast with an Evalue less than E^−20^) than genes on the autosomes (Pearson’s Chi-squared test with Yates’ continuity correction *P* = 0.003).

## Supplementary Material

Supporting Information
