# Supplementary material for: Widespread Selection Across Coding and Noncoding DNA in the Pea Aphid Genome
Source: G3 (Bethesda). 2013 Jun 1;3(6):993–1001. doi: 10.1534/g3.113.005793 (PMC3689810; doi:10.1534/g3.113.005793)
Supplement: Supporting Information [file supp_3_6_993__index.html]

Widespread Selection Across Coding and Noncoding DNA in the Pea Aphid Genome — Supporting Information 

# Widespread Selection Across Coding and Noncoding DNA in the Pea Aphid Genome

## Supporting Information for Bickel, Dunham, and Brisson, 2013

**Files in this Data Supplement:**

- Table S1 - Coverage per line (.xlsx, 38 KB)
- Table S2 - Primers and restriction enzymes used for RFLP analysis to confirm whether a scaffold was X-linked (X) or autosomal (A). (.xlsx, 39 KB)
- Table S3 - Fst values for each gene (.xlsx, 967 KB)
- Table S4 - GO overrepresentation analysis for genes with top 10% Tajima's D values (.xlsx, 357 KB)
- Table S5 - Probability of being on the X chromosome for each genomic scaffold (.xlsx, 71 KB)
